# Prevalence and risk factors for postextubation dysphagia in ICU patients with orotracheal intubation: a systematic review and meta-analysis

**DOI:** 10.3389/fmed.2026.1810274

**Published:** 2026-04-30

**Authors:** Ziwei He, Xinru Song, Aijian Lei, Yanxin Ma, Yuding Hu, Xiao Li, Cheng Zhang, Pingping Gao, Yanan Cao, Fei Tong, Guoying Wang

**Affiliations:** 1 Department of Intensive Care Medicine, The Second Hospital of Hebei Medical University, Shijiazhuang, Hebei, China; 2 Nursing School of Hebei Medical University, Shijiazhuang, Hebei, China; 3 Department of Emergency, The Second Hospital of Hebei Medical University, Shijiazhuang, Hebei, China

**Keywords:** intensive care unit, meta-analysis, postextubation dysphagia, prevalence, risk factors

## Abstract

**Background:**

Evidence suggests that 41% of ICU patients acquired postextubation dysphagia, substantially increasing the risk of aspiration and malnutrition. Studies on its prevalence and risk factors showed considerable variation. Our study aims to summarize the overall prevalence and identify risk factors for postextubation dysphagia in ICU patients undergoing orotracheal intubation.

**Methods:**

We searched PubMed, Embase, Web of Science, Cochrane Library, CINAHL, Medline, China National Knowledge Infrastructure, Wanfang, SinoMed, and Technology Journal Database for studies on postextubation dysphagia from inception to July 5, 2025. Two researchers independently conducted the literature screening, quality assessment, and extracted data. Meta-analysis was performed using Stata software 18.0 and Review Manager software 5.3.

**Results:**

Twenty-five studies were included, encompassing a total of 7,219 patients. The meta-analysis revealed that the overall prevalence of postextubation dysphagia was 35% (95% CI: 25–46). Age (OR = 1.03), age ≥ 65 years (OR = 2.72), age ≥ 70 years (OR = 2.34), Acute Physiology and Chronic Health Evaluation II (APACHE II) score (OR = 1.29), APACHE II score ≥ 15 points (OR = 4.69), arrhythmia (OR = 3.30), neurological disorders (OR = 3.77), tracheal intubation duration in hours (OR = 1.03), tracheal intubation duration in days (OR = 1.13), tracheal intubation duration ≥72 h (OR = 8.15), tracheal intubation duration ≥7 days (OR = 2.06), gastric tube retention (OR = 6.59), and gastric tube retention duration ≥72 h (OR = 3.43), emergency admission (OR = 2.30) were risk factors for postextubation dysphagia.

**Conclusion:**

The incidence of PED in ICU patients is relatively high, which is influenced by various factors. Based on the identified risk factors, clinical staff can early identify high-risk individuals and implement targeted preventive measures to avoid postextubation dysphagia.

**Systematic review registration:**

https://www.crd.york.ac.uk/PROSPERO/, CRD420251090144.

## 1 Introduction

Postextubation dysphagia (PED) is defined as the difficulty or inability to effectively and safely transfer food and liquid from the mouth to the stomach after extubation (1). Worldwide, 13 to 20 million critically ill patients require invasive mechanical ventilation annually for respiratory support (2). Orotracheal intubation is a common invasive mechanical ventilation method for critically ill patients in the intensive care unit (ICU), which is an important contributor to PED. PED can lead to aspiration, aspiration pneumonia, delayed oral intake, and subsequent malnutrition (3–5), which result in prolonged hospital stays and increased healthcare costs (3). Moreover, PED is an independent predictor for death at both 28 and 90 days among ICU patients (6). Determining the incidence and identifying risk factors for PED is of great significance for taking preventive measures and improving the prognosis of patients (7).

The reported incidence of PED varies greatly in different studies, ranging from 3 to 62% (3), and even some reports suggest it can reach 80% (8). An earlier Meta-analysis reported a combined incidence of PED at 41% (3), while another updated review reported it at 36% (8). Although meta-analyses have explored risk factors for PED, the results have been inconsistent. For example, a meta-analysis found that gender and intubation duration were not significant risk factors for PED (9), while another study pointed out that age, female gender, intubation duration, etc., are risk factors (10). It is necessary to conduct a new systematic review and meta-analysis to integrate both PED prevalence and its risk factors into a single study, while providing more comprehensive evidence for clinical practice.

Thereby, this study made a systematic review and meta-analysis with the latest evidence to summarize the incidence and risk factors of PED in ICU Patients with orotracheal intubation, providing valuable insights for future research on PED.

## 2 Methods

### 2.1 Reporting and registration protocol

This study adhered to the Preferred Reporting Items for Systematic Reviews and Meta-Analyses (PRISMA) guidelines (11). The review was registered with the International Prospective Register of Systematic Review (CRD420251090144).

### 2.2 Eligibility criteria

The inclusion criteria were: (1) patients in ICU undergoing orotracheal extubation; patients aged ≥18 years; (2) risk factors related to PED in ICU patients with orotracheal intubation; (3) patients who received an evaluation with at least one swallowing-specific assessment tools; (4) the study design includes randomized controlled trial, case–control study, cohort study and cross-sectional study; (5) the language was Chinese or English. The exclusion criteria included (1) duplicate publication; (2) unavailability of the complete text, or incomplete data where the necessary data could not be extracted or converted into OR and 95% CI; (3) low-quality studies.

### 2.3 Search strategy

A thorough search was conducted across PubMed, Embase, Web of Science, Cochrane Library, CINAHL, Medline, China National Knowledge Infrastructure (CNKI), Wanfang, SinoMed, and Technology Journal Database (VIP) from inception to July 5, 2025. This study adhered to the PICOS principle, and we used a combination of Medical Subject Headings (MeSH) and free-text terms, including “intensive care units/critical illness/critical care/ICU,” “airway extubation/artificial respiration,” “deglutition disorders/deglutition/dysplasia,” and “risk factors/influence factors.” The search strategy was appropriately adjusted for each database’s requirements. Manual retrieval of references from relevant articles was conducted to ensure comprehensive coverage. The whole search strategy is provided in Supplementary material 1.

### 2.4 Study selection

The search results were imported into EndNote 21. Following systematic and manual deduplication, two researchers independently screened the articles and crosschecked them. After screening the titles and abstracts, the researchers removed any articles that failed to meet the criteria. Then, the researchers read the full texts of studies that seemed to meet the inclusion and exclusion criteria. Disagreements were resolved by a third researcher.

### 2.5 Quality assessment

We assessed risk of bias in randomized controlled trials using the revised Cochrane tool (RoB2) (12), which graded each domain as high, low, or unclear risk. We evaluated the cohort and case–control studies using the Newcastle-Ottawa Scale (NOS) (13), which comprises three dimensions. The studies were assigned to a scale in which 0–3 represented low quality, 4–6 moderate, and 7–9 high quality. We assessed cross-sectional studies using the quality evaluation criteria recommended by the Agency for Healthcare Research and Quality (AHRQ) (14). The quality assessments included 11 items, categorized as low, moderate, or high, corresponding to scores of 0–3, 4–7, and 8–11, respectively. The quality of the included studies was independently evaluated by two researchers. Discussion or consultation was used with a third researcher to settle all disputes.

### 2.6 Data extraction

Data were extracted by reviewers using Microsoft Excel. The extracted data included: author, country, year of publication, study design, sample size, swallowing assessment tools, timing of assessment, incidence, risk factors, and effect sizes. Discussion or consultation was made with a third researcher to solve all disagreements.

### 2.7 Data analysis

All acquired data were recorded in Excel for preprocessing. The combined prevalence of PED was calculated using Stata software 18.0, and incidence estimates were transformed using the “Freeman-Tukey Double Arcsine Transformation” to approximate the normal distribution of the data. The meta-analysis of risk factors was conducted using Review Manager software 5.3. The OR values and their 95% CI for each risk factor were used as the effect size. *I^2^* was chosen to assess heterogeneity across the included studies. *I^2^* ≤ 50% and *p* ≥ 0.1 indicate less heterogeneity among studies; the fixed-effects model was used for the meta-analysis. Otherwise, it indicated high heterogeneity. Subgroup analyses or the one-by-one elimination method are conducted to identify the source of significant heterogeneity. If the heterogeneity remains substantial, we use the random-effects model. Sensitivity analyses of the article are performed using both the one-by-one elimination method and the transformation effects model to assess the study’s stability. Funnel plots and Egger’s tests were performed using Stata software 18.0 to assess publication bias in PED incidence. The trim-and-fill method was used to identify and correct potential publication bias. Statistical significance was defined as a *p*-value of <0.05.

## 3 Results

### 3.1 Search results

**Figure 1:**
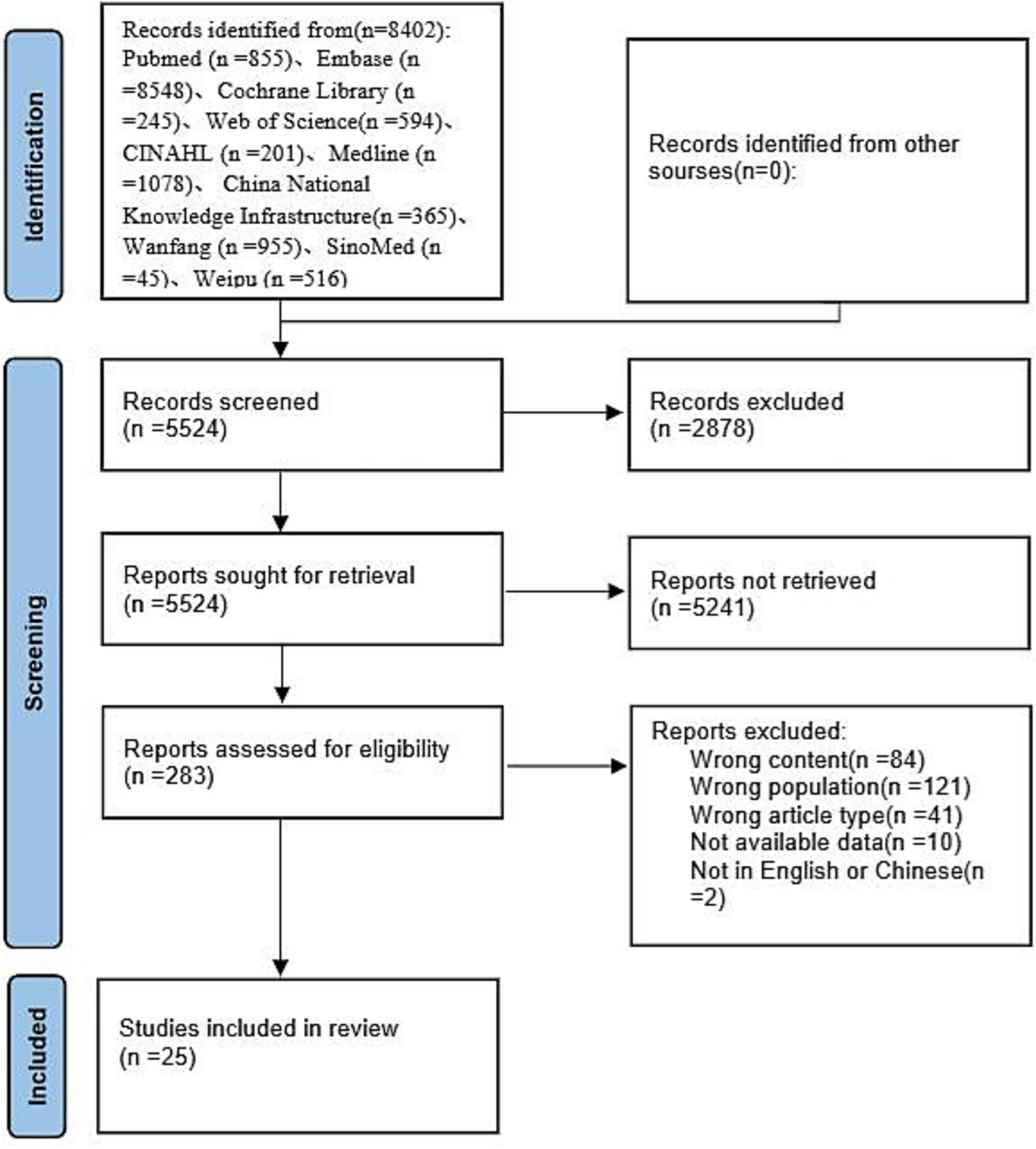
Literature screening flowchart.

In all, 8,402 articles were retrieved through database searches. After removing duplicate references, 5,524 articles were obtained. Subsequently, 283 articles remained after screening titles and abstracts. After a full-text review according to the criteria, 25 articles were eventually included. The process of literature screening is presented in Figure 1.

### 3.2 Characteristics and quality evaluation of the included studies

The basic characteristics and methodological quality evaluation of the included studies are summarized in Table 1. In total, 7,219 participants were enrolled in 25 studies, comprising 17 cohort studies (6, 15–30), 4 case–control studies (31–34), and 4 cross-sectional studies (5, 35–37). The incidence of PED in the included studies ranged from 4 to 84%. The methods for assessing PED vary across studies. The methodological quality of the evaluation scores for the included 25 studies was all ≥ 6 points, indicating high quality (Supplementary material 1).

**Table 1:** Summarized characteristics and quality evaluation of included studies.

Study	Country	Study design	Study population	Sample	Assessment tool	Assessment time	Incidence of PED (%)	Risk factor	Quality (score)
Wan et al. (2018) (31)	China	Case–control study	Respiratory ICU Surgical ICU	154	SSA	4 h after extubation	36	APACHE II score, tracheal intubation duration, arrhythmia	7
Guo et al. (2020a) (15)	China	Cohort study	Surgical ICU Mixed medical and surgical ICU	152	SSA	4–6 h after extubation	26	APACHE II score, tracheal intubation duration, age, gastric tube retention	9
Guo (2020b) (16)	China	Cohort study	Surgical ICU Mixed medical and surgical ICU	238	SSA	4–6 h after extubation	27	APACHE II score, tracheal intubation duration, arrhythmia, age, gastric tube retention	9
Ji (2020) (32)	China	Case–control study	Mixed medical and surgical ICU	222	SSA	After extubation	46	Tracheal intubation duration, age	7
Deng et al. (2021) (17)	China	Cohort study	ICU	180	SSA	4 h after extubation	40	APACHE II score, tracheal intubation duration, age	8
Pan (2022) (35)	China	Cross-sectional study	Mixed medical and surgical ICU	267	SSA	4–6 h after extubation	68	Gastric tube retention, gender, neurologic disease	10
Cao and Wang (2023) (36)	China	Cross-sectional study	ICU	180	SSA	Not stated	10	APACHE II score, tracheal intubation duration, age	8
Shao et al. (2023) (18)	China	Cohort study	Cardiac ICU	387	SSA	4 h after extubation	8	Tracheal intubation duration, age, gastric tube retention	8
Jiang and Zhang (2024) (33)	China	Case–control study	Cardiac ICU	274	SSA	Within 24 h after extubation	29	APACHE II score, tracheal intubation duration, length of ICU stay	9
Wang et al. (2024) (34)	China	Case–control study	ICU	214	GuSS-ICU	6 h after extubation	30	APACHE II score, tracheal intubation duration, age	9
Hogue et al. (1995) (19)	US	Cohort study	Respiratory ICU	869	Barium swallow test	After extubation	4	Tracheal intubation duration, age	9
Solh et al. (2003) (20)	US	Cohort study	ICU	84	FEES	Within 48 h after extubation	44	Age	9
Barker et al. (2009) (21)	Canada	Cohort study	Cardiac ICU	254	BSE → VFFS	After extubation	51	Tracheal intubation duration	7
Bordon et al. (2011) (22)	US	Cohort study	Trauma ICU	150	WST	Within 24 h after extubation	41	Tracheal intubation duration, age	7
Macht et al. (2011) (23)	US	Cohort study	ICU	446	BSE	Not stated	84	Tracheal intubation duration	7
Kwok et al. (2013) (24)	US	Cohort study	Trauma ICU	239	CSE	Within 24 h after extubation	47	Tracheal intubation duration, age	8
Brodsky et al. (2014) (25)	US	Cohort study	Respiratory ICU	132	SSQ	After discharge	34	Tracheal intubation duration	8
Tsai et al. (2016) (26)	China	Cohort study	ICU	151	BSE	Within 48 h after extubation	61	Age	8
Scheel et al. (2016) (37)	US	Cross-sectional study	Mixed medical and surgical ICU	59	FEES	After extubation	58	Emergent admission	7
Schefold et al. (2017) (6)	Switzerland	Cohort study	Mixed medical and surgical ICU	933	WSTs	Within 3 h after extubation	12	APACHE II score, neurologic disease, emergency admission	9
Oliveira et al. (2018) (5)	Brazil	Cross-sectional study	ICU	181	MASA	Within 48 h after extubation	36	Age	9
Zuercher et al. (2020) (27)	Switzerland	Cohort study	Mixed medical and surgical ICU	933	BSE	Within 3 h after extubation	12	APACHE II score, tracheal intubation duration, age, gender, neurologic disease, emergency admission	7
Zeng et al. (2021) (28)	China	Cohort study	Neurosurgical ICU	206	SSA	Within 24 h after extubation	23	APACHE II score, tracheal intubation duration, gastric tube retention, length of ICU stay	7
Maamar et al. (2022) (29)	France	Cohort study	Mixed medical and surgical ICU	79	BSSD→FEES	Within 24 h after extubation	42	Tracheal intubation duration	8
Tang et al. (2023) (30)	China	Cohort study	Emergency ICU Central ICU Cardiac ICU	235	SSA	4–6 h after extubation	23	Age, gastric tube retention	7

SSA, standardized swallowing assessment; GUSS-ICU, Gugging Swallowing Screen for the Intensive Care Unit; FEES, fiberoptic endoscopic evaluation of swallowing; VEES, videofluoroscopic swallowing study; BSE, bedside swallowing evaluation; WST/WSTs, water swallow test; CSE, clinical swallowing evaluation; SSQ, Sydney Swallowing Questionnaire; MASA, Mann Assessment of Swallowing Ability; BSSD, bedside screening test of swallowing dysfunction.

### 3.3 Meta-analysis results

#### 3.3.1 The incidence of PED

**Figure 2:**
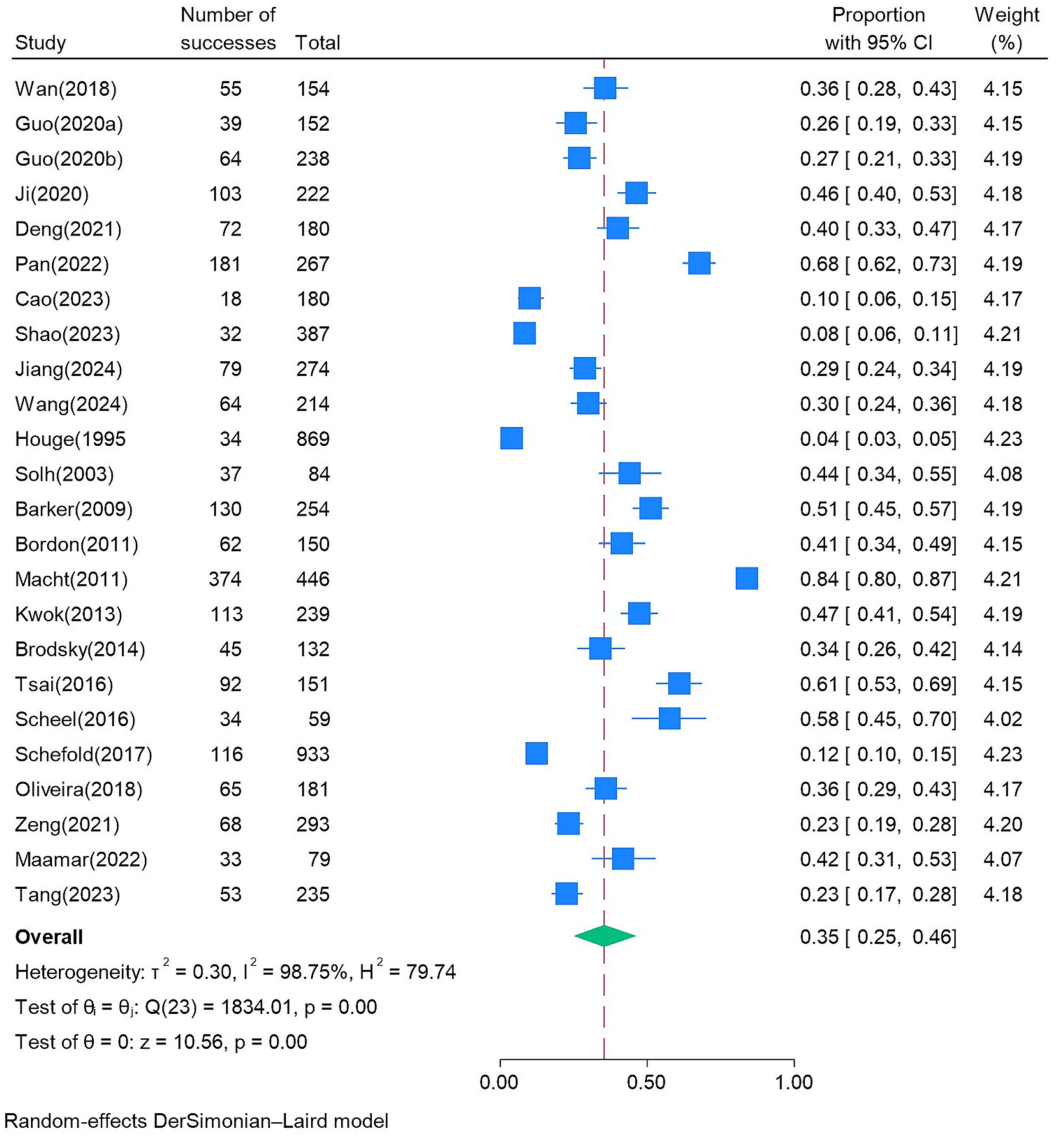
Forest plot of the incidence of PED.

Due to the studies by Schefold et al. (6) and Zuercher et al. (27), which used data from the same database and reported the same PED incidence, the incidence from 24 studies was ultimately used for the Meta-analysis. There was a high degree of heterogeneity among the included studies (*I^2^* = 98.75%, *p* < 0.001), so the random-effects model was used for the meta-analysis. The meta-analysis results showed that the pooled incidence of PED was 35% (95% CI: 25–46), as shown in Figure 2. The subgroup analysis of the included studies was conducted according to the assessment tool, assessment time, study region, study type, and ICU type. The results of the subgroup analysis of PED prevalence in patients are shown in Table 2 (Supplementary material 2). Then, after eliminating each study in sequence, a sensitivity analysis was conducted. The results showed that the overall prevalence of PED did not change significantly, suggesting that the results were relatively stable (Supplementary material 2).

**Table 2:** Subgroup analysis of the incidence of PED.

Subgroup	Classification	Number of studies^a^	Heterogeneity		Effects model	Meta-analysis (%)	
			*I^2^* (%)	*p*		Incidence	95% CI
Assessment tool	Instrument assessment	5	99.04	<0.001	Random	37	10–70
	Swallowing test	18	98.64	<0.001		35	24–47
Assessment time	<24 h after extubation	14	97.42	<0.001	Random	31	22–41
	>24 h after extubation	4	89.13	<0.001		44	31–57
Study region	America	9	99.33	<0.001	Random	43	20–69
	Asia	13	97.31	<0.001		32	22–42
	Europe	2	97.10	<0.001		25	3–58
Study type	Case–control study	4	84.40	<0.001	Random	35	27–43
	Cohort study	16	99.03	<0.001		34	21–48
	Cross-sectional study	4	98.39	<0.001		41	14–71
ICU type	Cardiac ICU	3	98.75	<0.001	Random	27	6–56
	Mixed medical and surgical ICU	5	98.96	<0.001		44	18–72
	Respiratory ICU	2	98.79	0.07		16	0–54
	Trauma ICU	2	23.41	<0.001		45	39–51

a The number of studies varies as not all studies addressed each subgroup.

#### 3.3.2 Risk factors of PED

Although Schefold et al. (6) and Zuercher et al. (27) used data from the same database, the risk factors were different, so both studies were included in the meta-analysis. We conducted a meta-analysis of risk factors reported in two or more studies (Supplementary material 3). For variables such as age, APACHE II score, duration of tracheal intubation, the studies included both continuous variables and categorical variables. We conducted meta-analyses for continuous variables and categorical variables independently.

Data on age from one study (19), on emergency admission from one study (37) and on tracheal intubation duration from two studies (19, 21) were incomplete and could not be included in the combined analysis. The meta-analysis revealed that age (OR = 1.03, 95% CI: 1.02–1.05), age ≥ 65 years (OR = 2.72, 95% CI: 1.84–4.00), age ≥ 70 years (OR = 2.34, 95% CI: 1.41–3.89), APACHE II score (OR = 1.29, 95% CI: 1.14–1.45), APACHE II score ≥ 15 points (OR = 4.69, 95% CI: 2.53–8.70), arrhythmia (OR = 3.30, 95% CI: 1.53–7.14), and neurological disorders (OR = 3.77, 95% CI: 2.76–5.15), tracheal intubation duration in hours (OR = 1.03, 95% CI: 1.01–1.04), tracheal intubation duration in days (OR = 1.13, 95% CI: 1.09–1.16), tracheal intubation duration ≥72 h (OR = 8.15, 95% CI: 4.85–13.70), tracheal intubation duration ≥7 days (OR = 2.06, 95% CI: 1.18–3.58), gastric tube retention (OR = 6.59, 95% CI: 2.56–16.93), and gastric tube retention duration ≥72 h (OR = 3.43, 95% CI: 1.85–6.34), emergency admission (OR = 2.30, 95% CI: 1.54–3.45) were risk factors for PED (Table 2). Gender (OR = 2.07, 95% CI: 0.82–5.26), ICU length of stay (OR = 2.34, 95% CI: 0.83–6.57) were not correlated with PED. Additionally, two studies (32, 33) evaluated the association between heart rate during extubation and PED. One study (33) analyzed heart rate as a continuous variable, whereas the other (32) reported it as a binary variable. Two studies (5, 30) indicated that the risk of PED increased in ICU patients with voice disorders. However, their definitions of abnormal voice and assessment tools varied, and no raw data were provided for conversion. Therefore, we cannot perform a meta-analysis on the two factors mentioned above.

Transforming the fixed-effects and random-effects model to evaluate the included risk factors, the results showed that tracheal intubation duration in hours, gender, and ICU length of stay were unstable in the combined analysis of the altered data analysis models, which may be related to fewer studies included and high heterogeneity. The results of the meta-analysis of other risk factors showed no significant changes, indicating that the results of this study remained relatively consistent, as shown in Table 3.

**Table 3:** Risk factors and meta-analysis results of PED.

Risk factors	*N*	*I^2^*	*p*	Effects model	Pooled OR (95% CI)	Sensitivity analysis OR (95% CI)
Age (5, 18, 24)	3	0	0.50	Fixed	1.03 (1.02, 1.05)*	1.03 (1.02, 1.05)* stable
Age ≥ 65 years (15, 16, 20, 26)	4	26	0.25	Fixed	2.72 (1.84, 4.00)*	2.75 (1.74, 4.34)* stable
Age ≥ 70 years (32, 36)	2	40	0.20	Fixed	2.34 (1.41, 3.89)*	2.54 (1.22, 5.25)* stable
Gender (27, 35)	2	81	0.02	Random	2.07 (0.82, 5.26)	1.88 (1.27, 2.80)* unstable
APACHE II score (6, 17, 27, 28, 31, 33)	6	93	<0.001	Random	1.29 (1.14, 1.45)*	1.08 (1.06, 1.11)* stable
APACHE II score ≥ 15 points (15, 16)	2	0	0.91	Fixed	4.69 (2.53, 8.70)*	4.69 (2.53, 8.70)* stable
Arrhythmia (16, 31)	2	0	0.63	Fixed	3.30 (1.53, 7.14)*	3.30 (1.53, 7.14)* stable
Neurological disorders (6, 27, 35)	3	0	0.65	Fixed	3.77 (2.76, 5.15)*	3.77 (2.76, 5.15)* stable
Tracheal intubation in hours (18, 28, 33)	3	35	0.22	Fixed	1.03 (1.01, 1.04)*	1.03 (1.00, 1.06) unstable
Tracheal intubation in days (22, 27, 29)	3	41	0.19	Fixed	1.13 (1.09, 1.16)*	1.12 (1.08, 1.18)* stable
Tracheal intubation duration ≥ 72 h (15, 16, 36)	3	0	0.78	Fixed	8.15 (4.85, 13.70)*	8.15 (4.85, 13.70)* stable
Tracheal intubation duration ≥ 7 days (23, 34)	2	78	0.03	Random	2.06 (1.18, 3.58)*	1.80 (1.46, 2.23)* stable
Gastric tube retention (18, 28, 30)	3	0	0.86	Fixed	6.59 (2.56, 16.93)*	6.59 (2.56, 16.93)* stable
Gastric tube retention duration ≥ 72 h (15, 16)	2	0	0.98	Fixed	3.43 (1.85, 6.34)*	3.43 (1.85, 6.34)* stable
Emergency admission (6, 27)	2	0	0.56	Fixed	2.30 (1.54, 3.45)*	2.30 (1.54, 3.45)* stable
ICU length of stay (28, 33)	2	90	0.002	Random	2.34 (0.83, 6.57)	1.63 (1.31, 2.02)* unstable

OR, odds ratio; CI, confidence interval; APACHEII, Acute Physiology and Chronic Health Evaluation II; *N*, number of included studies; *p < 0.05.

The sensitivity analysis was conducted through one-by-one elimination. Sensitivity analysis of the APACHE II score did not find the source of heterogeneity. Factors such as age, age ≥70 years, APACHE II ≥ 15 points, tracheal intubation duration in hours, and tracheal intubation duration in days with high heterogeneity were analyzed by sequentially excluding each study. After removing the study that contributed to heterogeneity, the fixed-effects model was used. Studies were excluded mainly because of study design, patient characteristics, and assessment methods, etc. These exclusions reduced *I^2^* values, improving the robustness of the pooled analyses and the interpretability of the results while preserving the original evidence base. A summary of the excluded studies is illustrated in Supplementary material 3.

### 3.4 Publication bias

Funnel plots of studies reporting prevalence exhibited slight asymmetry. Egger’s test showed that *t* = 3.82, *p* = 0.001, indicating publication bias in this study (Supplementary material 2). Following publication bias correction using the trim and fill method (3 missing studies imputed), the pooled prevalence of PED increased, suggesting that our original results might have been underestimated.

## 4 Discussion

### 4.1 Incidence of PED

The study results show that the overall incidence of PED in ICU patients with oral intubation is 35%. This result is largely consistent with a recent review (8) reporting an incidence of 36%, but is slightly lower than the 41% reported by McIntyre et al. (3) earlier. The difference may stem from updates to the included studies and from variations in the methods used to handle heterogeneity. This study found extremely high heterogeneity among the studies (*I^2^* = 98.75%). Despite the subgroup analysis, this highly heterogeneous source still exists. The issue may be attributed to variations in assessment methods and patients’ characteristics (38).

We employed 10 different tools to assess PED across the studies in this review,yet the subgroup results indicated that the assessment tool did not reduce heterogeneity among the studies. The subgroup analysis revealed that the incidence of PED was higher in studies based on the instrument assessment used to assess PED, at 37%. This is largely because instruments such as VFSS and FEES are regarded as the gold standard for diagnosing dysphagia (39), and they can detect dysphagia with high sensitivity. However, they need professional personnel and equipment, resulting in high costs. In contrast, clinical swallowing tests are simple to operate and easy to master. The incidence of PED among the patients who were evaluated 24 h after extubation was 44%, which was higher than that of the patients evaluated within 24 h after extubation. This difference may be attributed to the study by Schefold et al. (6) and Shao et al. (18), which had the shorter intubation time. Future prospective studies should employ standardized tools for longitudinal, multi-time-point assessments to determine the trajectory of PED over time.

Differences in the prevalence of PED can be attributed to variations in ICU type. The subgroups consisted of respiratory, cardiac, trauma, and mixed medical and surgical ICUs. We found that the incidence of PED was the highest among trauma ICU patients, and only the statistical heterogeneity in this type was reduced. The pooled incidence was 16% (95% CI: 0–54) in the respiratory ICU, and the difference was not statistically significant. It suggests that the decision to assess PED should not be based solely on ICU type. Additionally, the incidence of PED in different regions also varies. The reported incidence in America is higher than in Asia and Europe, which may be related to the greater use of VFSS and FEES in North America. The difference in study types may also be a cause for the varied incidences. All in all, future studies should focus on establishing unified assessment criteria for PED and conducting high-quality studies to enhance the comparability of the results.

### 4.2 Risk factors of PED

This study conducted a meta-analysis to comprehensively and systematically explore risk factors for PED by integrating the latest evidence. The results revealed that age, APACHE II score, arrhythmia, neurological disorders, tracheal intubation duration, gastric tube retention, and emergency admission were risk factors for PED.

Regarding patient factors, age is a key risk factor for PED, and the risk of PED increases continuously with age. The findings of the meta-analysis show that the risk of PED in patients aged ≥65 years is 2.72 times that of younger patients. Even though the age standard is raised to 70 years or above, the risk of PED in these patients is still 2.34 times that of younger patients. This risk comparison is slightly lower in the ≥65-year-old group. This may be related to Ji et al. (32), which had a relatively high weight in the meta-analysis but reported a lower risk ratio. This result indicates that at different age levels, age is a stable and significant risk factor for PED. This may be associated with the physiological degenerative changes accompanying the elderly (40). With age, elderly patients often have multiple underlying diseases and may experience atrophy of the swallowing muscles (41) and decreased tongue pressure (42). Under stress and traumatic operations such as tracheal intubation, the swallowing function is more likely to be damaged (43). Therefore, medical staff should focus on elderly patients who have been extubated, especially those aged 65 or older. They should carry out systematic swallowing function assessment as soon as possible and implement targeted swallowing function rehabilitation training based on individual circumstances in order to reduce the incidence of PED.

Regarding disease factors, the APACHE II score is a scoring system widely used for ICU patients, which comprehensively assesses the acute physiological and chronic health status of critically ill patients (44). A high APACHE II score indicates a more severe condition in the patient, and such patients have a poorer prognosis (43). They usually require a longer intubation time, which can further impair the swallowing function. Arrhythmias like atrial fibrillation can promote atrial thrombus formation, which can then embolize to the brain and cause an ischemic stroke (10). While neurological disorders such as stroke and brain trauma can damage the central and peripheral nerve pathways that control swallowing, resulting in a decrease in swallowing function (28). Thereby, medical staff should strengthen screening and intervention for swallowing function in patients with a high APACHE II score and a history of arrhythmia or neurological disorder. These people should actively treat the primary diseases and improve patients’ nutritional status.

Regarding treatment factors, the meta-analysis shows that patients with tracheal intubation duration ≥ 72 h have an 8.15 times higher risk of PED than those with < 72 h of intubation. In comparison, patients with intubation duration ≥ 7 days have a 2.06 times higher risk of PED than those with <7 days The former has a higher risk of PED, which could be linked to the fact that the patients of Cao et al. (36) were elderly people, and this group has lower physiological reserves. Even in a shorter intubation period, they are more prone to PED. Tracheal intubation can directly damage the throat mucosa, leading to local edema, inflammation, decreased sensory function, and impaired swallowing coordination (45–47). Long-term intubation will further cause atrophy of the muscles in the tongue, pharynx, and larynx (48), increasing the risk of PED. The meta-analysis shows that patients with gastric tube retention duration ≥ 72 h have a 3.43 times higher risk of PED than those with short-term gastric tube retention. The reason may be that continuous compression and stimulation of the stomach tube on the pharynx lead to pharyngeal edema, sensory loss, and inhibition of the swallowing reflex (49). And long-term non-oral feeding will cause atrophy of swallowing-related muscles and oral hygiene problems, affecting the swallowing function of ICU patients (49). Therefore, in cases where the patient’s condition permits, high-risk individuals should be evaluated and trained for swallowing function as early as possible, and gradually resume oral feeding to promote recovery of swallowing function.

Patients with emergency admission demonstrate a higher risk of PED. Patients with emergency admission are usually in a more severe condition, and their intubation sites are usually outside the hospital or in the emergency department (10). During the operation, they are more prone to swallowing-related neuromuscular damage (10), further increasing the risk of swallowing dysfunction.

In addition, this study has the following limitations: First, only Chinese and English studies were retrieved, and no randomized controlled trials were included, which could impact the thoroughness of the study results. Second, some risk factors were derived from only 2 to 3 studies, which may introduce bias into the results. Third, data on some factors were not feasible to combine because of insufficient research quantity and significant differences in classification criteria, and we were unable to analyze their relationship with PED. Fourth, the included studies differed in assessment tool, timing, and patient population, which may have contributed to the heterogeneity in this study. It is recommended that future research continue to explore the incidence and risk factors of PED and conduct more large-sample, multi-center, high-quality studies.

Despite the limitations above, our findings had significant clinical implications. This study revealed a significant prevalence of PED and ascertained risk factors for PED. Moreover, Schefold et al. (6) found that neurological diseases and emergency admission were not only risk factors for PED, but also closely related to the recovery process of patients’ swallowing function. The latest study (50) further revealed different trajectories of swallowing function recovery, suggesting that patients’ rehabilitation processes exhibit significant individual differences. Therefore, it is recommended to standardize assessment tools, timing, and screening procedures for PED in ICU patients. At the same time, high-quality longitudinal studies should be conducted in the future to deeply explore the recovery trajectory of PED, construct a risk prediction model for swallowing function recovery, and provide a basis for identifying early high-risk groups and formulating rehabilitation measures.

## 5 Conclusion

This meta-analysis provides important insights into the overall prevalence of PED and its related risk factors. PED is a common complication, occurring in 35% of critically ill patients after orotracheal intubation. There are numerous risk factors for PED, highlighting the urgent need for early identification and preventive measures to reduce the incidence of PED. The above conclusions require further validation and improvement through larger-sample, multi-center, high-quality studies.

## Data Availability

The original contributions presented in the study are included in the article/Supplementary material, further inquiries can be directed to the corresponding authors.
