# Correction: Prevalence and risk factors for postextubation dysphagia in ICU patients with orotracheal intubation: a systematic review and meta-analysis

**DOI:** 10.3389/fmed.2026.1900284

**Published:** 2026-06-16

**Authors:** Frontiers Production Office

**Affiliations:** Frontiers Media SA, Lausanne, Switzerland

**Keywords:** intensive care unit, meta-analysis, postextubation dysphagia, prevalence, risk factors

Authors Ziwei He and Xinru Song should be recognized as co-first authors. Both authors made equal contributions to this manuscript.

Ziwei He^1,2†^, Xinru Song^1,2†^, Aijian Lei^1^, Yanxin Ma^1^, Yuding Hu^1^, Xiao Li^3^, Cheng Zhang^1^, Pingping Gao^1^, Yanan Cao^1^, Fei Tong^1*^ and Guoying Wang^1*^

^†^These authors have contributed equally to this work

The original version of this article has been updated.

