# Supplementary material for: Prevalence and risk factors for postextubation dysphagia in ICU patients with orotracheal intubation: a systematic review and meta-analysis
Source: Front Med (Lausanne). 2026 Apr 30;13:1810274. doi: 10.3389/fmed.2026.1810274 (PMC13171512; doi:10.3389/fmed.2026.1810274)
Supplement: Supplementary file 1 [file Data_Sheet_1.DOCX]

Search Strategy

**PubMed**

#1 ((((((((Critical Illness[MeSH Terms]) OR (Critical Care[MeSH Terms])) OR (Intensive Care Units[MeSH Terms])) OR (critical illness[Title/Abstract])) OR (critical care[Title/Abstract])) OR (critical ill[Title/Abstract])) OR (intensive care unit*[Title/Abstract])) OR (intensive care[Title/Abstract])) OR (ICU[Title/Abstract])

#2 ((((((((((((((Intubation, Intratracheal[MeSH Terms]) OR (Airway Extubation[MeSH Terms])) OR (Respiration, Artificial[MeSH Terms])) OR (ventilators, mechanical[MeSH Terms])) OR (artificial respiration[MeSH Terms])) OR (respiration,artificial[Title/Abstract])) OR (airway extubation[Title/Abstract])) OR (ventilators, mechanical[Title/Abstract])) OR (mechanical ventilation*[Title/Abstract])) OR (artificial ventilation[Title/Abstract])) OR (artificial airway[Title/Abstract])) OR (intubation[Title/Abstract])) OR (extubation[Title/Abstract])) OR (intratracheal[Title/Abstract])) OR (endotracheal[Title/Abstract])

#3 ((((((((Deglutition Disorders[MeSH Terms])) OR (deglutition[Title/Abstract])) OR (deglutition disorder*[Title/Abstract])) OR (swallowing disorder*[Title/Abstract])) OR (swallow*[Title/Abstract])) OR (dysphagi*[Title/Abstract])) OR (oropharyngeal dysphagia[Title/Abstract])) OR (esophageal dysphagia[Title/Abstract])

#4 (((((((Risk Factors[MeSH Terms]) OR (risk factor*[Title/Abstract])) OR (factor*[Title/Abstract])) OR (influence factor*[Title/Abstract])) OR (associated factor*[Title/Abstract])) OR (relevant factor*[Title/Abstract])) OR (predicted factor*[Title/Abstract])) OR (reason*[Title/Abstract])

#5 (#1 OR #2) AND #3 AND #4

**Embase**

#1 'intensive care unit'/exp

#2 'critical illness'/exp

#3 'intensive care'/exp

#4 'critical illness':ti,ab,kw OR 'critical care':ti,ab,kw OR 'critical ill':ti,ab,kw OR 'intensive care unit*':ti,ab,kw OR 'intensive care':ti,ab,kw OR icu:ti,ab,kw

#5 #1 OR #2 OR #3 OR #4

#6 'endotracheal intubation'/exp

#7 'extubation'/exp

#8 'mechanical ventilator'/exp

#9 'artificial ventilation'/exp

#10 'intratracheal intubation':ti,ab,kw OR 'endotracheal intubation':ti,ab,kw OR 'airway extubation':ti,ab,kw OR 'artificial respiration':ti,ab,kw OR 'ventilators, mechanical':ti,ab,kw OR 'mechanical ventilation*':ti,ab,kw OR 'artificial ventilation':ti,ab,kw OR 'artificial airway':ti,ab,kw OR intubation:ti,ab,kw OR extubation:ti,ab,kw OR intratracheal:ti,ab,kw OR endotracheal:ti,ab,kw

#11 #6 OR #7 OR #8 OR #9 OR #10

#12 'dysphagia'/exp

#13 'deglutition:ti,ab,kw OR 'deglutition disorder*':ti,ab,kw OR 'swallowing disorder*':ti,ab,kw OR swallow*:ti,ab,kw OR dysphagi*:ti,ab,kw OR 'oropharyngeal dysphagia':ti,ab,kw OR 'esophageal dysphagia':ti,ab,kw

#14 #12 OR #13
#15 'risk factor'/exp

#16 'risk factor*':ti,ab,kw OR factor*:ti,ab,kw OR 'influence factor*':ti,ab,kw OR 'associated factor*':ti,ab,kw OR 'relevant factor*':ti,ab,kw OR 'predicted factor*':ti,ab,kw OR reason*:ti,ab,kw

#17 #15 OR #16

#18 (#5 OR #11) AND #14 AND #17

**Web of science**

#1 Topic= (critical illness OR critical care OR critical ill OR intensive care unit* OR intensive care OR ICU)

#2 Topic=(intratracheal intubation OR airway extubation OR artificial respiration OR ventilators, mechanical OR mechanical ventilation* OR artificial ventilation OR artificial airway OR intubation OR extubation OR intratracheal OR endotracheal)

#3 Topic=(deglutition OR deglutition disorder* OR swallowing disorder* OR swallow* OR dysphagi* OR oropharyngeal dysphagia OR esophageal dysphagia)

#4 Topic=(risk factor* OR factor* OR influence factor* OR associated factor* OR relevant factor* OR predicted factor* OR reason*)

#5 (#1 OR #2) AND #3 AND #4

**Cochrane library**

#1 MeSH descriptor: [Intensive Care Units] explode all trees

#2 MeSH descriptor: [Critical Illness] explode all trees

#3 MeSH descriptor: [Critical Care] explode all trees

#4 (critical illness OR critical care OR critical ill OR intensive care unit* OR intensive care OR ICU):ti,ab,kw

#5 #1 OR #2 OR #3 OR #4

#6 MeSH descriptor: [Intubation, Intratracheal] explode all trees

#7 MeSH descriptor: [Airway Extubation] explode all trees

#8 MeSH descriptor: [Respiration, Artificial] explode all trees

#9 MeSH descriptor: [Ventilators, Mechanical] explode all trees

#10 (intratracheal intubation OR airway extubation OR artificial respiration OR ventilators, mechanical OR mechanical ventilation* OR artificial ventilation OR artificial airway OR intubation OR extubation OR intratracheal OR endotracheal):ti,ab,kw

#11 #6 OR #7 OR #8 OR #9 OR #10

#12 MeSH descriptor: [Deglutition Disorders] explode all trees

#13 (deglutition OR deglutition disorder* OR swallowing disorder* OR swallow* OR dysphagi* OR oropharyngeal dysphagia OR esophageal dysphagia):ti,ab,kw

#14 #12 OR #13

#15 MeSH descriptor: [Risk Factors] explode all trees

#16 (risk factor* OR factor* OR influence factor* OR associated factor* OR relevant factor* OR predicted factor* OR reason*):ti,ab,kw

#17 #15 OR #16

#18 (#5 OR #11) AND #14 AND #17

**CINAHL**

#1 SU=critical illness OR critical care OR critical ill OR intensive care unit* OR intensive care OR ICU

#2 SU=intratracheal intubation OR airway extubation OR artificial respiration OR mechanical ventilation* OR artificial ventilation OR artificial airway OR intubation OR extubation OR intratracheal OR endotracheal

#3 SU=deglutition OR deglutition disorder* OR swallowing disorder* OR swallow* OR dysphagi* OR oropharyngeal dysphagia OR esophageal dysphagia

#4 SU=risk factor* OR factor* OR influence factor* OR associated factor* OR relevant factor* OR predicted factor* OR reason*

#5 (#1 OR #2) AND #3 AND #4

**Medline**

#1 Topic= (critical illness OR critical care OR critical ill OR intensive care unit* OR intensive care OR ICU)

#2 Topic=(intratracheal intubation OR airway extubation OR artificial respiration OR ventilators, mechanical OR mechanical ventilation* OR artificial ventilation OR artificial airway OR intubation OR extubation OR intratracheal OR endotracheal)

#3 Topic=(deglutition OR deglutition disorder* OR swallowing disorder* OR swallow* OR dysphagi* OR oropharyngeal dysphagia OR esophageal dysphagia)

#4 Topic=(risk factor* OR factor* OR influence factor* OR associated factor* OR relevant factor* OR predicted factor* OR reason*)

#5 (#1 OR #2) AND #3 AND #4

**CNKI**

#1主题=重症监护室 OR 重症监护病房 OR 重症 OR 危重症 OR 监护室 OR 加强护理病房 OR ICU

#2 主题=气管插管 OR 机械通气 OR 人工气道 OR 呼吸机 OR 拔管

#3 主题=吞咽障碍 OR 吞咽困难 OR 进食障碍 OR 咽下困难 OR 吞咽功能

#4 主题=危险因素 OR 影响因素 OR 风险因素 OR 相关因素 OR 预测因素 OR 因素

OR 原因

#5 (#1 OR #2) AND #3 AND #4

Wanfang

#1主题=重症监护室 OR 重症监护病房 OR 重症 OR 危重症 OR 监护室 OR 加强护理病房 OR ICU

#2 主题=气管插管 OR 机械通气 OR 人工气道 OR 呼吸机 OR 拔管

#3 主题=吞咽障碍 OR 吞咽困难 OR 进食障碍 OR 咽下困难 OR 吞咽功能

#4 主题=危险因素 OR 影响因素 OR 风险因素 OR 相关因素 OR 预测因素 OR 因素 OR 原因

#5 (#1 OR #2) AND #3 AND #4

SinoMed

#1 "重症监护病房"[不加权:扩展]

#2 ( "重症监护室"[常用字段:智能] OR "重症监护病房"[常用字段:智能] OR "重症"[常用字段:智能] OR "危重症"[常用字段:智能] OR "监护室"[常用字段:智能] OR "加强护理病房"[常用字段:智能] OR "ICU"[常用字段:智能])

#3 #1 OR #2

#4 "气管插管拔除"[不加权:扩展]

#5 ("气管插管"[常用字段:智能] OR "机械通气"[常用字段:智能] OR "人工气道"[常用字段:智能] OR "呼吸机"[常用字段:智能] OR "拔管"[常用字段:智能] OR "气管插管拔除"[常用字段:智能])

#6 #4 OR #5

#7 "吞咽障碍"[不加权:扩展]

#8 ( "吞咽困难"[常用字段:智能] OR "进食障碍"[常用字段:智能] OR "咽下困难"[常用字段:智能] OR "吞咽功能"[常用字段:智能])

#9 #7 OR #8

#10 "危险因素"[不加权:扩展]

#11 ( "影响因素"[常用字段:智能] OR "风险因素"[常用字段:智能] OR "相关因素"[常用字段:智能] OR "预测因素"[常用字段:智能] OR "因素"[常用字段:智能] OR "原因"[常用字段:智能])

#12 #10 OR #11

#13 (#3 OR #6) AND #9 AND #12

**VIP**

#1题名或关键词=重症监护室 OR 重症监护病房 OR 重症 OR 危重症 OR 监护室 OR 加强护理病房 OR ICU

#2 题名或关键词=气管插管 OR 机械通气 OR 人工气道 OR 呼吸机 OR 拔管

#3 题名或关键词=吞咽障碍 OR 吞咽困难 OR 进食障碍 OR 咽下困难 OR 吞咽功能

#4 题名或关键词=危险因素 OR 影响因素 OR 风险因素 OR 相关因素 OR 预测因素 OR 因素 OR 原因

#5 (#1 OR #2) AND #3 AND #4

**Quality assessment**

**Supplementary Table 1** Quality assessment of case-control studies

| Study | Selection | Comparability | Outcome | Total score |
| --- | --- | --- | --- | --- |
| Wan,2018(1) | 3 | 2 | 2 | 7 |
| Ji,2020(2) | 3 | 2 | 2 | 7 |
| Jiang,2024(3) | 4 | 2 | 3 | 9 |
| Wang,2024(4) | 4 | 2 | 3 | 9 |

**Supplementary Table 2** Quality assessment of cohort studies

| Study | Selection | Comparability | Outcome | Total score |
| --- | --- | --- | --- | --- |
| Guo,2020a(5) | 4 | 2 | 3 | 9 |
| Guo,2020b(6) | 4 | 2 | 3 | 9 |
| Deng,2021(7) | 4 | 2 | 2 | 8 |
| Shao,2023(8) | 4 | 2 | 2 | 8 |
| Hogue,1995(9) | 4 | 2 | 3 | 9 |
| Solh,2003(10) | 4 | 2 | 3 | 9 |
| Barker,2009(11) | 3 | 1 | 3 | 7 |
| Bordon,2011(12) | 3 | 1 | 3 | 7 |
| Macht,2011(13) | 3 | 2 | 2 | 7 |
| Kwok,2013(14) | 3 | 2 | 3 | 8 |
| Brodsky,2014(15) | 4 | 2 | 2 | 8 |
| Tsai,2016(16) | 4 | 1 | 3 | 8 |
| Schefold,2017(17) | 4 | 2 | 3 | 9 |
| Zuercher,2020(18) | 4 | 2 | 1 | 7 |
| Zeng,2021(19) | 4 | 2 | 1 | 7 |
| Maamar,2022(20) | 3 | 2 | 3 | 8 |
| Tang,2023(21) | 4 | 2 | 1 | 7 |

**Supplementary Table 3** Quality assessment of cross-sectional studies

| Study | (1) | (2) | (3) | (4) | (5) | (6) | (7) | (8) | (9) | (10) | (11) | Total score |
| --- | --- | --- | --- | --- | --- | --- | --- | --- | --- | --- | --- | --- |
| Pan,2022(22) | Yes | Yes | Yes | Yes | Yes | Yes | Yes | Yes | Unclear | Yes | Yes | 10 |
| Cao,2023(23) | Yes | Yes | Yes | Yes | Yes | Yes | Unclear | Yes | Unclear | Yes | No | 8 |
| Scheel,2016(24) | Yes | Yes | Yes | Yes | Yes | Yes | Unclear | No | Unclear | Yes | No | 7 |
| Oliveira,2018(25) | Yes | Yes | Yes | Yes | Yes | Yes | Yes | Yes | Unclear | Yes | No | 9 |

(1) Define the source of information (survey, record review).

(2) List inclusion and exclusion criteria for exposed and unexposed subjects (cases and controls) or refer to previous publications.

(3) Indicate time period used for identifying patients.

(4) Indicate whether or not subjects were consecutive if not population-based.

(5) Indicate if evaluators of subjective components of study were masked to other aspects of the status of the participants.

(6) Describe any assessment undertaken for quality assurance purposes (e.g. test/retest of primary outcome measurements).

(7) Explain any patient exclusions from analysis.

(8) Describe how confounding was assessed and/ or controlled.

(9) If applicable, explain how missing data were handled in the analysis.

(10) Summarize patient response rates and completeness of data collection.

(11) Clarify what follow-up, if any, was expected and the percentage of patients for which incomplete data or follow-up obtained.

**References**

1. Wan N, Wang YL, Zhang CY, Wang SQ, JIia YR, Yang N, et al. The status and risk factors of acquired dysphagia in ICU patients. Chinese Nursing Management 2018;18(11).doi:103969/j.issn.1672-1756.2018.11.007

2. Ji ZS. Risk factors analysis and predictive model of postextubation swallowing dysfunction in ICU patients 2020.

3. Jiang W, Zhang W. Analysis of risk factors for dysphagia after extubation in patients with tracheal intubation in the CSICU. Chin J Emerg Resusc Disaster Med. 2024;19(5)

4. Wang LY, Sheng H, Bu HQ, Li MS. Analysis of risk factors for Dysphagia in Critically Ill Patients After tracheal extubation Zhejiang J Trauma Surg. 2024;29(9).doi:10.3969/j.issn.1009-7147.2024.09.031

5. Guo F, Wang MM, Zou SQ. Analysis of risk factors and establishment of prediction model for post ⁃ extubation swallowing dysfunction in ICU patients with endotracheal intubation. Chinese Nursing Reaserch. 2020;34(19).doi:10.12102/j.issn.1009-6493.2020.19.009

6. Guo F. Post-extubation dysphagia of patients with endotracheal intubation in ICU: risk factors analysis and prediction model establishment 2020.

7. Deng Y, Zhang Y, Ye YL. Risk factors and nursing strategies of the occurrences of acquired swallowing disorders after ICU patients treated with oral tracheal Intubation and extubation. Medical Equipment. 2021;34(1)

8. Shao AM, Xia HO, Zhong J, Zhang Q, Chen YH, Yuan X. Value of preoperative maXimum tongue pressure in predicting swallowing dysphagia after cardiac surgery. Journal of Nursing Science. 2023;38(11).doi:10.3870/j.issn.1001-4152.2023.11.023

9. Hogue CW, Jr., Lappas GD, Creswell LL, Ferguson TB, Jr., Sample M, Pugh D, et al. Swallowing dysfunction after cardiac operations. Associated adverse outcomes and risk factors including intraoperative transesophageal echocardiography. The Journal of thoracic and cardiovascular surgery. 1995;110(2):517-22.doi:10.1016/s0022-5223(95)70249-0

10. El Solh A, Okada M, Bhat A, Pietrantoni C. Swallowing disorders post orotracheal intubation in the elderly. Intensive care medicine. 2003;29(9):1451‐5.doi:10.1007/s00134-003-1870-4

11. Barker J, Martino R, Reichardt B, Hickey EJ, Ralph-Edwards A. Incidence and impact of dysphagia in patients receiving prolonged endotracheal intubation after cardiac surgery. Can J Surg. 2009;52(2):119-24

12. Bordon A, Bokhari R, Sperry J, Testa D, Feinstein A, Ghaemmaghami V. Swallowing dysfunction after prolonged intubation: analysis of risk factors in trauma patients. American Journal of Surgery. 2011;202(6):679-82.doi:10.1016/j.amjsurg.2011.06.030

13. Macht M, Wimbish T, Clark BJ, Benson AB, Burnham EL, Moss M. Dysphagia is common, persistent, and associated with poor outcomes in survivors of critical illness. American Journal of Respiratory and Critical Care Medicine. 2011;183(1)

14. Kwok AM, Davis JW, Cagle KM, Sue LP, Kaups KL. Post-extubation dysphagia in trauma patients: it's hard to swallow. Am J Surg. 2013;206(6):924-7; discussion 7-8.doi:10.1016/j.amjsurg.2013.08.010

15. Brodsky MB. Duration of oral endotracheal intubation is associated with dysphagia symptoms in acute lung injury patients. Journal of critical care. 2014;29(4):574-9

16. Tsai M-H, Ku S-C, Wang T-G, Hsiao T-Y, Lee J-J, Chan D-C, et al. Swallowing dysfunction following endotracheal intubation: Age matters. Medicine. 2016;95(23):e3871-e.doi:10.1097/MD.0000000000003871

17. Schefold JC. Dysphagia in Mechanically Ventilated ICU Patients (DYnAMICS): A Prospective Observational Trial. Critical care medicine. 2017;45(12):2061-9

18. Zuercher P. Risk Factors for Dysphagia in ICU Patients After Invasive Mechanical Ventilation. Chest. 2020;158(5):1983-91

19. Zeng L, Song Y, Dong Y, Wu Q, Zhang L, Yu L, et al. Risk Score for Predicting Dysphagia in Patients After Neurosurgery: A Prospective Observational Trial. Front Neurol. 2021;12:605687.doi:10.3389/fneur.2021.605687

20. Maamar A, Parent V, Prudhomme E, Guérot E, Berneau P, Frérou A, et al. Fiberoptic endoscopic validation of a clinical screening test of swallowing function in critically ill patients performed within 24 h after extubation. J Crit Care. 2022;72:154119.doi:10.1016/j.jcrc.2022.154119

21. Tang JY, Feng XQ, Huang XX, Zhang YP, Guo ZT, Chen L, et al. Development and validation of a predictive model for patients with post-extubation dysphagia. World journal of emergency medicine. 2023;14(1):49-55.doi:10.5847/wjem.j.1920-8642.2023.021

22. Pan XH. Analysis of swallowing function after extubation in patients with prolonged orotracheal intubation and its influencing factors: Soochow University; 2022.

23. Cao XZ, Wang RJ. Construction and validation of a risk prediction model for acquired dysphagia in elderly patients with mechanical ventilation in the ICU. Modern Nurse. 2023;30(12).doi:10.19792/j.cnki.1006-6411.2023.35.022

24. Scheel R, Pisegna JM, McNally E, Noordzij JP, Langmore SE. Endoscopic Assessment of Swallowing After Prolonged Intubation in the ICU Setting. Annals of Otology, Rhinology &amp; Laryngology. 2016;125(1):43-52.doi:10.1177/0003489415596755

25. Oliveira ACM, Friche AAL, Salomão MS, Bougo GC, Vicente LCC. Predictive factors for oropharyngeal dysphagia after prolonged orotracheal intubation. Braz J Otorhinolaryngol. 2018;84(6):722-8.doi:10.1016/j.bjorl.2017.08.010
