# Supplementary material for: Prevalence and risk factors for postextubation dysphagia in ICU patients with orotracheal intubation: a systematic review and meta-analysis
Source: Front Med (Lausanne). 2026 Apr 30;13:1810274. doi: 10.3389/fmed.2026.1810274 (PMC13171512; doi:10.3389/fmed.2026.1810274)
Supplement: Supplementary file 2 [file Data_Sheet_2.docx]

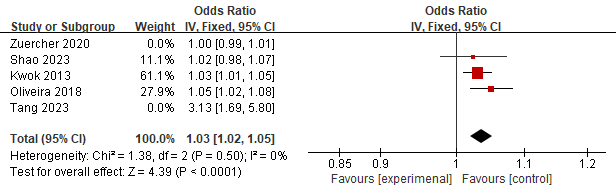


Supplementary Figure. 1 Forest plots of age

The figure shows a statistically significant association between age and PED, with a combined OR of 1.03 (95% CI: 1.02-1.05).


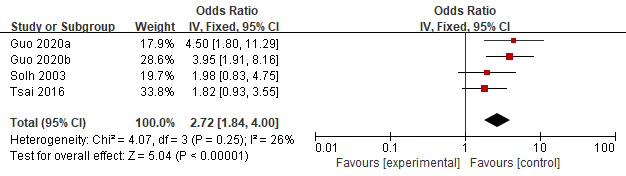


Supplementary Figure. 2 Forest plots of age ≥65 years

The figure shows a statistically significant association between age ≥65 years and PED, with a combined OR of 2.72 (95% CI: 1.84-4.00).


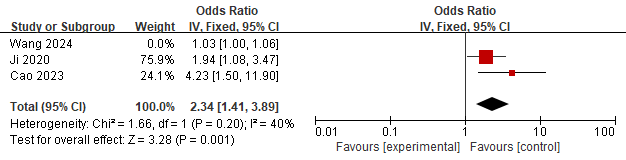


Supplementary Figure. 3 Forest plots of age ≥70 years

The figure shows a statistically significant association between age ≥70 years and PED, with a combined OR of 2.34 (95% CI: 1.41-3.89).


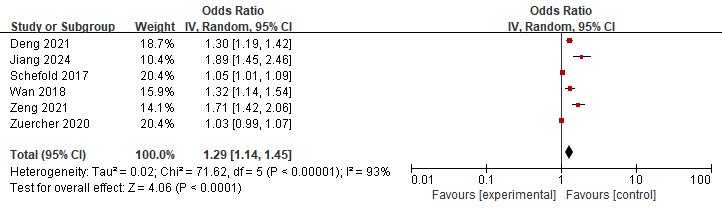


Supplementary Figure. 4 Forest plots of APACHE Ⅱ score

The figure shows a statistically significant association between APACHE Ⅱ score and PED, with a combined OR of 1.29 (95% CI: 1.14-1.45).


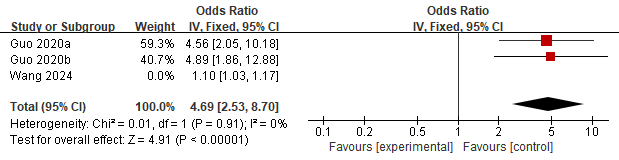


Supplementary Figure. 5 Forest plots of APACHE Ⅱ score ≥15 points

The figure shows a statistically significant association between APACHE Ⅱ score ≥15 points and PED, with a combined OR of 4.69 (95% CI: 2.53-8.70).


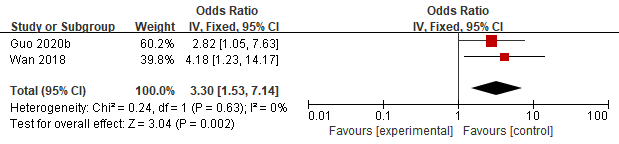


Supplementary Figure. 6 Forest plots of arrhythmia

The figure shows a statistically significant association between arrhythmia and PED, with a combined OR of 3.30 (95% CI: 1.53-7.14).


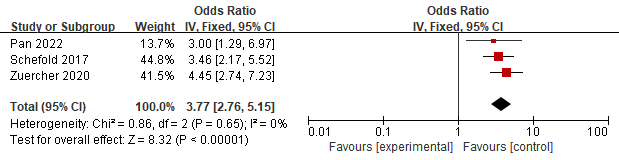


Supplementary Figure. 7 Forest plots of neurological disorders

The figure shows a statistically significant association between neurological disorders and PED, with a combined OR of 3.77 (95% CI: 2.76-5.15).


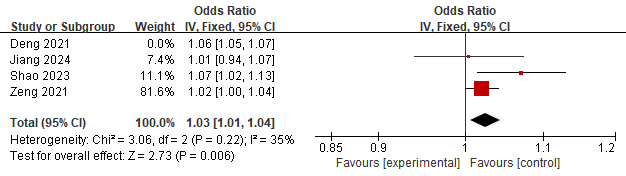


Supplementary Figure. 8 Forest plots of tracheal intubation in hours

The figure shows a statistically significant association between tracheal intubation in hours and PED, with a combined OR of 1.03 (95% CI: 1.01-1.04).


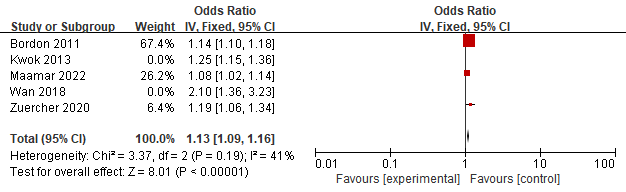


Supplementary Figure. 9 Forest plots of tracheal intubation in days

The figure shows a statistically significant association between tracheal intubation in days and PED, with a combined OR of 1.13 (95% CI: 1.09-1.16).


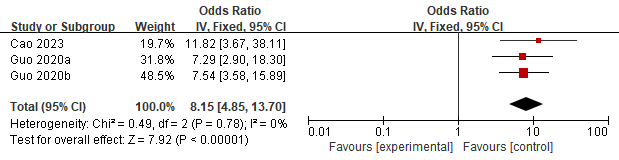


Supplementary Figure. 10 Forest plots of intubation duration ≥72 hours

The figure shows a statistically significant association between intubation duration ≥72 hours and PED, with a combined OR of 8.15 (95% CI: 4.85-13.70).


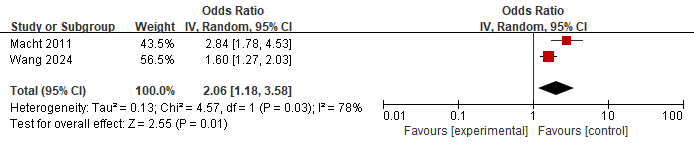


Supplementary Figure. 11 Forest plots of intubation duration ≥7 days

The figure shows a statistically significant association between intubation duration ≥7 days and PED, with a combined OR of 2.06 (95% CI: 1.18-3.58).


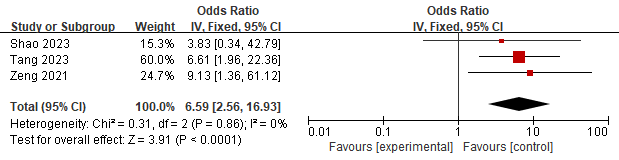


Supplementary Figure. 12 Forest plots of gastric tube retention

The figure shows a statistically significant association between gastric tube retention and PED, with a combined OR of 6.59 (95% CI: 2.56-16.93).


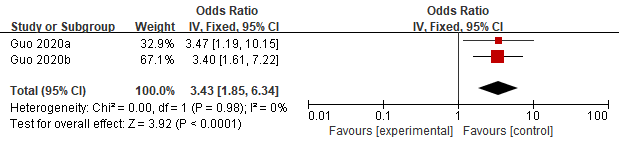


Supplementary Figure. 13 Forest plots of gastric tube retention duration ≥72 hours

The figure shows a statistically significant association between gastric tube retention duration ≥72 hours and PED, with a combined OR of 3.43 (95% CI: 1.85-6.34).


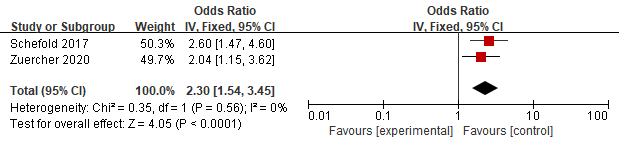


Supplementary Figure. 14 Forest plots of emergency admission

The figure shows a statistically significant association between emergency admission and PED, with a combined OR of 2.30 (95% CI: 1.54-3.45).


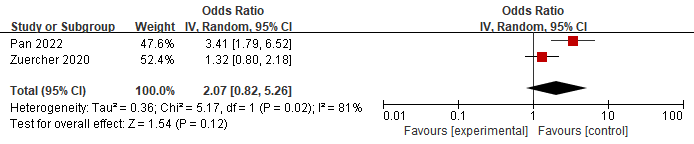


Supplementary Figure. 15 Forest plots of gender

The figure shows no statistically significant association between gender and PED, with a combined OR of 2.07 (95% CI: 0.82-5.26).


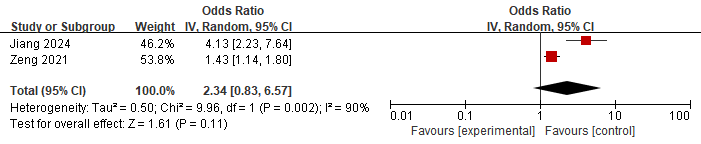


Supplementary Figure. 16 Forest plots of ICU length of stay

The figure shows no statistically significant association between ICU length of stay and PED, with a combined OR of 2.34 (95% CI: 0.83-6.57).

Figure1-16 caption: Each study corresponds to a horizontal line and a square. The size of the square represents the weight of the study in the pooled analysis, and the length of the horizontal line represents the 95% CI. The pooled fixed-effect estimate and its 95% CI are represented by a dashed vertical and a diamond.

[Table 1](https://pmc.ncbi.nlm.nih.gov/articles/PMC12515452/#t0005). Summary of the excluded studies

| **Risk factors** | **Number of excluded studies** | **Number of studies included after elimination** |
| --- | --- | --- |
| Age | 2(1, 2) | 3 |
| Age ≥70 years | 1(3) | 2 |
| APACHE Ⅱ score ≥15 points | 1(3) | 2 |
| Tracheal intubation duration in hours | 1(4) | 3 |
| Tracheal intubation duration in days | 2(5, 6) | 3 |

**References**

1. Zuercher P. Risk Factors for Dysphagia in ICU Patients After Invasive Mechanical Ventilation. Chest. 2020;158(5):1983-91

2. Tang JY, Feng XQ, Huang XX, Zhang YP, Guo ZT, Chen L, et al. Development and validation of a predictive model for patients with post-extubation dysphagia. World journal of emergency medicine. 2023;14(1):49-55.doi:10.5847/wjem.j.1920-8642.2023.021

3. Wang LY, Sheng H, Bu HQ, Li MS. Analysis of risk factors for Dysphagia in Critically Ill Patients After tracheal extubation Zhejiang J Trauma Surg. 2024;29(9).doi:10.3969/j.issn.1009-7147.2024.09.031

4. Deng Y, Zhang Y, Ye YL. Risk factors and nursing strategies of the occurrences of acquired swallowing disorders after ICU patients treated with oral tracheal Intubation and extubation. Medical Equipment. 2021;34(1)

5. Wan N, Wang YL, Zhang CY, Wang SQ, JIia YR, Yang N, et al. The status and risk factors of acquired dysphagia in ICU patients. Chinese Nursing Management 2018;18(11).doi:103969/j.issn.1672-1756.2018.11.007

6. Kwok AM, Davis JW, Cagle KM, Sue LP, Kaups KL. Post-extubation dysphagia in trauma patients: it's hard to swallow. Am J Surg. 2013;206(6):924-7; discussion 7-8.doi:10.1016/j.amjsurg.2013.08.010
