# Supplementary material for: Prevalence and risk factors for postextubation dysphagia in ICU patients with orotracheal intubation: a systematic review and meta-analysis
Source: Front Med (Lausanne). 2026 Apr 30;13:1810274. doi: 10.3389/fmed.2026.1810274 (PMC13171512; doi:10.3389/fmed.2026.1810274)
Supplement: Supplementary file 3 [file Supplementary_File_1.docx]

Supplementary Figure 1 Subgroup analysis of the incidence of PED by assessment tool

This figure presents a subgroup analysis of the incidence of PED based on the assessment tool. Due to the high heterogeneity among the included studies (*I^2^* = 98.80%), the random-effects model was used. The subgroup analysis results showed that the pooled incidence in the instrument assessment group was 37% (95% CI: 10-70), while in the swallowing test group was 35% (95% CI: 24- 47).

Caption: Each study corresponds to a horizontal line and a square. The size of the square represents the weight of the study in the pooled analysis, and the length of the horizontal line represents the 95% CI. The pooled fixed-effect estimate and its 95% CI are represented by a dashed vertical and a diamond.

Supplementary Figure 2 Subgroup analysis of the incidence of PED by assessment time

This figure presents a subgroup analysis of the incidence of PED based on the assessment time. Due to the high heterogeneity among the included studies (*I^2^* = 97.16%), the random-effects model was used. The subgroup analysis results showed that the pooled incidence in the assessment time < 24h after extubation group was 31% (95% CI: 22-41), while in the > 24h after extubation group was 44% (95% CI: 31- 57).

Caption: Each study corresponds to a horizontal line and a square. The size of the square represents the weight of the study in the pooled analysis, and the length of the horizontal line represents the 95% CI. The pooled fixed-effect estimate and its 95% CI are represented by a dashed vertical and a diamond.

Supplementary Figure 3 Subgroup analysis of the incidence of PED by study region

This figure presents a subgroup analysis of the incidence of PED based on the study region. Due to the high heterogeneity among the included studies (*I^2^* = 98.75%), the random-effects model was used. The subgroup analysis results showed that the pooled incidence in the America group was 43% (95% CI: 20-69), in the Asia group was 32% (95% CI: 22-42), in the Europe group was 25% (95% CI: 3-58).

Caption: Each study corresponds to a horizontal line and a square. The size of the square represents the weight of the study in the pooled analysis, and the length of the horizontal line represents the 95% CI. The pooled fixed-effect estimate and its 95% CI are represented by a dashed vertical and a diamond.

Supplementary Figure 4 Subgroup analysis of the incidence of PED by study type

This figure presents a subgroup analysis of the incidence of PED based on the study type. Due to the high heterogeneity among the included studies (*I^2^* = 98.75%), the random-effects model was used. The subgroup analysis results showed that the pooled incidence in the case-control study group was 35% (95% CI: 27-43), in the cohort study group was 34% (95% CI: 21-48), in the cross-sectional study group was 41% (95% CI: 14-71).

Caption: Each study corresponds to a horizontal line and a square. The size of the square represents the weight of the study in the pooled analysis, and the length of the horizontal line represents the 95% CI. The pooled fixed-effect estimate and its 95% CI are represented by a dashed vertical and a diamond.

Supplementary Figure 5 Subgroup analysis of the incidence of PED by ICU type

This figure presents a subgroup analysis of the incidence of PED based on the ICU type. Due to the high heterogeneity among the included studies (*I^2^* = 98.87%), the random-effects model was used. The subgroup analysis results showed that the pooled incidence in the cardiac ICU group was 27% (95% CI: 6-56), in the mixed medical and surgical ICU group was 44% (95% CI: 18-72), in the respiratory ICU group was 16% (95% CI: 0-54), in the trauma ICU group was 45% (95% CI:39-51)

Caption: Each study corresponds to a horizontal line and a square. The size of the square represents the weight of the study in the pooled analysis, and the length of the horizontal line represents the 95% CI. The pooled fixed-effect estimate and its 95% CI are represented by a dashed vertical and a diamond.

Supplementary Figure 6 Sensitivity analysis of the incidence of PED

This figure presents the results of a meta-analysis of combined incidence after eliminating individual studies one by one. Due to the high heterogeneity, the random-effects model was used. The sensitivity analysis showed that after eliminating any single study, the pooled incidence remained stable within the range of 33% to 37%. This indicates that the results of this meta-analysis are relatively robust and reliable.

Caption: Each dot represents the combined incidence rate of the remaining studies after eliminating the corresponding study. The length of the horizontal line represents the 95% CI.

Supplementary Figure 7 Funnel plot of the incidence of PED

This figure presents the funnel plot of the incidence of PED. It exhibits slight asymmetry.

Caption: Each study corresponds to a dot.

Supplementary Figure 8 Funnel plots after correction by trim and fill method

This figure presents the funnel plots after correction by trim and fill method. Following the imputation of 3 missing studies, the pooled prevalence of PED increased, indicating that our original results might have been underestimated.

Caption: Each study corresponds to a dot.

Supplementary Figure 9 Egger's test of the incidence of PED

This figure presents the Egger's tests. The results showed that t = 3.82, *p* = 0.001, indicating that there might be publication bias in this study.

Caption: Each study corresponds to a dot.
